# How Can Artificial Intelligence Be Implemented Effectively in Diabetic Retinopathy Screening in Japan?

**DOI:** 10.3390/medicina60020243

**Published:** 2024-01-30

**Authors:** Ryo Kawasaki

**Affiliations:** 1 Division of Public Health, Department of Social Medicine, Graduate School of Medicine, Osaka University, Suita 565-0871, Japan; rkawasaki@pbhel.med.osaka-u.ac.jp; 2 Artificial Intelligence Center for Medical Research and Application, Osaka University Hospital, Suita 565-0871, Japan

**Keywords:** diabetic retinopathy, artificial intelligence, systematic screening, large language models

## Abstract

Diabetic retinopathy (DR) is a major microvascular complication of diabetes, affecting a substantial portion of diabetic patients worldwide. Timely intervention is pivotal in mitigating the risk of blindness associated with DR, yet early detection remains a challenge due to the absence of early symptoms. Screening programs have emerged as a strategy to address this burden, and this paper delves into the role of artificial intelligence (AI) in advancing DR screening in Japan. There are two pathways for DR screening in Japan: a health screening pathway and a clinical referral path from physicians to ophthalmologists. AI technologies that realize automated image classification by applying deep learning are emerging. These technologies have exhibited substantial promise, achieving sensitivity and specificity levels exceeding 90% in prospective studies. Moreover, we introduce the potential of Generative AI and large language models (LLMs) to transform healthcare delivery, particularly in patient engagement, medical records, and decision support. Considering the use of AI in DR screening in Japan, we propose to follow a seven-step framework for systematic screening and emphasize the importance of integrating AI into a well-designed screening program. Automated scoring systems with AI enhance screening quality, but their effectiveness depends on their integration into the broader screening ecosystem. LLMs emerge as an important tool to fill gaps in the screening process, from personalized invitations to reporting results, facilitating a seamless and efficient system. However, it is essential to address concerns surrounding technical accuracy and governance before full-scale integration into the healthcare system. In conclusion, this review highlights the challenges in the current screening pathway and the potential for AI, particularly LLM, to revolutionize DR screening in Japan. The future direction will depend on leadership from ophthalmologists and stakeholders to address long-standing challenges in DR screening so that all people have access to accessible and effective screening.

## 1. Introduction

Diabetic retinopathy (DR) is a common microvascular complication of diabetes, with an estimated prevalence of about one in four diabetic patients [1]. The International Diabetes Federation (IDF) estimated that the number of diabetic patients worldwide will reach 700 million by 2045 [2]. Although some reports indicate that the risk of diabetic patients developing new DR is decreasing, the number of patients with sight-threatening DR (i.e., proliferative diabetic retinopathy (PDR) OR diabetic macular edema (DME)) is estimated to reach 44.82 million by 2045 [3].

Significant advances have been made in the treatment of DR over the past 30 years, and timely treatment can greatly reduce the risk of blindness or severe and irreversible visual impairment. However, there are still cases of irreversible vision loss, and the reasons for this include the lack of subjective symptoms in the early stages of DR, which delays the detection of DR. In the shadow of progress in DR care, the remaining challenge is to detect DR early enough to secure access to effective treatment. The disease burden of diabetes and DR remains heavy, and early detection of DR by screening programs is one of the strategies to counteract this burden [4]. 

This article aims to provide a review of the impact of artificial intelligence (AI) on automated diagnosis models and other potential use cases. In considering the use and challenges of using AI-automated diagnostic models for DR, this paper first provides an overview of screening for DR from a historical perspective, then describes the types of screening for which AI-automated diagnostic models have been developed, and finally describes how AI automated diagnostic models can be used for screening for DR in Japan. Finally, we will discuss how large language models (LLMs) can be used for the management and total healthcare of DR in Japan.

## 2. Why DR Needs to Be Screened, and How?

As shown in Table 1, diabetic retinopathy meets the criteria for screening for DR. Since the St. Vincent Declaration (1990) to the Liverpool Declaration (2005) and further conferences on screening for diabetic retinopathy in Europe [5], the Liverpool Declaration encouraged European countries to establish systematic screening to reduce the risk of visual impairment due to diabetic retinopathy by 2010 by (1) systematic programs of screening reaching at least 80% of the population with diabetes; (2) using trained professionals and personnel; and (3) universal access to laser therapy. Further meetings have been held, and it has been recommended that screening for DR be shifted from opportunistic screening to systemic screening, from unorganized to organized systematic screening, which has started to be implemented in some countries.

An example of systematic screening implemented country-wide is the screening program of the National Health Service (NHS) in the United Kingdom [6]. This is based on the strategy from the St. Vincent declaration, a four-stage concept that advocates the development of stepwise screening for DR (Table 2).

A path to establish systematic or organized screening consists of five further components, as presented in Table 3. Following these components, Japan’s situation can be reviewed below:

### 2.1. Screening Pathway

*“A pathway for DR screening should be in place, rather than the test being carried out in isolation”.*

**Figure 1:**
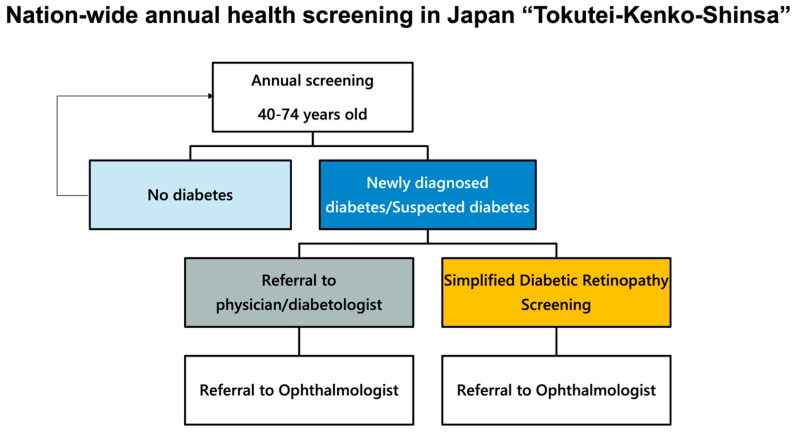
Two pathways of screening for diabetic retinopathy (DR) in Japan.

In Japan, current DR screening is provided two-fold (Figure 1); the first pathway is the national health screening program, and the second path is a referral from physicians to ophthalmologists. The first pathway is built into the national systematic screening program to detect diabetes in Japan, called the specific health checkups (“Tokutei-Kenko-Shinsa”) [7]. This is a nation-wide program for persons aged 40–74 years old under health insurance and includes a screening for diabetes, hypertension, dyslipidemia, and obesity.

### 2.2. Guidelines

*“The pathway is governed by protocols and guidelines”.*

The first pathway of the national systematic screening program for diabetes is conducted as the national screening program. This screening scheme is refined every 5 years, and the screening for DR has been recommended since the revision in 2018. There are no official grading protocols for DR defined in the health screening programs. However, there have been grading manuals and suggested recommendations published by the Ministry of Health, Labour, and Welfare [8] (which adopts a classification of simple DR, pre-proliferative DR, and PDR), the Japanese Society of Cardiovascular Disease Prevention [9], and the Japan Society for Ningen Dock [10] (these two adopt the international classification [11]). For the second pathway, the clinical guideline for DR in Japan recommends that when a patient is diagnosed with diabetes, persons without DR with mild to moderate NPDR, severe NPDR, and PDR should be screened annually, every 6 months, every 2 months, and monthly, respectively [12].

### 2.3. Quality Standard

*“There are quality standards based on evidence that service providers follow”.*

There are no quality standards for the two pathways of DR screening in Japan. The first path of DR screening in the national screening program is not mandatory, and no clear quality standards are set. The second pathway of referral from physicians to ophthalmologists is recommended, but no standards are defined. As a result of this opportunistic approach, the uptake of DR screening is estimated to remain low.

For the first pathway, the national health checkups (specific health checkups) are clearly defined by law in terms of subjects, examination methods, examination items, and guidance and recommendations based on the results of the checkups. Currently, for those who have diabetes or are suspected to have diabetes, fundus examinations are conducted as an optional examination for early detection of diabetic retinopathy. However, only informal guidelines are provided on how to perform the test, how to determine the test results, and how to provide guidance and recommendations on the test results.

In this pathway, standardization of technical specifications for fundus photography screening would be necessary to implement quality standard assurance. Specifically, the specification of the photographic fields (e.g., number of fields, degree of image size, the minimum image resolution of digital photographs, the file storage format, and the image viewing terminal settings) should be included in these specifications. In addition, training of judges and monitoring of intra-judges’ agreement rate, inter-judges’ agreement, and inter-institutional agreement as part of judgment accuracy management are essential for systematic examination accuracy management. The evaluation of outcomes, such as the rate of continuation of treatment, the rate of treatment implementation, and the visual prognosis, is also essential.

For the second pathway of clinical referral, there are no quality indicators to monitor the referral uptake between physicians and ophthalmologists. Since the national health insurance system data are gathered from the National Database (NDB) in Japan, there is a possibility that referral uptake can be monitored based on the health claims information [13].

### 2.4. Information System and Monitoring

*“The screening pathway is supported by an information system that can monitor performance”.*

There is no dedicated information system or monitoring system for both DR screening pathways. Japan has a national-level health insurance scheme, and the National Database (NDB) captures more than 95% of the screening linked to this health insurance scheme and clinical claims in Japan. Because DR screening is not a mandatory component of the health screening program, reported numbers are not useful to monitor screening uptake. For the second path of clinical referrals, Ihana-Sugiyama et al. reported that only half of the patients who are under treatment for diabetes had fundus examinations conducted in the NDB database [13].

In summary, Japan has DR screening in place via two pathways as a part of the national screening for diabetes and referrals between physicians and ophthalmologists; they are both still unsystematic and predominantly carried out by motivated ophthalmologists and health organizations. Although it was not discussed in this manuscript in detail, there is secured access to ophthalmologists (10.35 ophthalmologists per 100,000 population) and DR treatment in Japan. It remains a need to provide policymakers and stakeholders in the health insurance and health screening programs with the importance and challenges of DR screening so that more systematic screening can be offered in Japan.

## 3. Emerging AI Technologies That Can Be Applied to DR Screening

### 3.1. Image Classification for Diagnostic Support

To realize systematic screening, which requires a large amount of grading workload, many projects have been attempting to automate DR screening since the 1990s. Early approaches to automated screening used image processing filters so that they could process images step-by-step, e.g., first, image processing to exclude areas of non-retinopathy lesions (e.g., optic nerve papillae and blood vessels). Secondly, in the process of detecting red lesions (e.g., retinal hemorrhages) and yellow/white lesions (e.g., hard white spots and soft white spots), those attempts have achieved sensitivity and specificity at 80% and 80%, respectively [14].

A milestone that changed the situation was a series of studies using deep learning to classify color fundus images to identify DR in 2016 [15]. As an automatic judgment model for fundus images using deep learning, it was trained based on screening images and judgment results performed in the past and reached a level of sensitivity and specificity exceeding 90% at the same time, which became a hot topic. Subsequently, as a prospective study, the accuracy was verified at several hospitals, and it was reported that the sensitivity and specificity of approximately 90% could be maintained [16].

There is domestic research and companies that use automated grading of color fundus photographs using deep learning to assist in the diagnostic support of DR in Japan. DeepEyeVision Inc. (Tokyo, Japan) [17] has developed the original system and started a commercial service. This service aims to serve mainly for fundus photograph evaluation in the national health screening program.

Many other research projects and commercial services have been reported to date, and Table 4 lists those that have been prospectively verified in actual cases. All of them are now at high levels of both sensitivity and specificity and can achieve sensitivity ≥ 80% and specificity ≥ 90%.

### 3.2. Generative AI and Large Language Models

Since the release of ChatGPT (OpenAI, San Francisco, CA, USA) in 2022, large language models (LLMs) have revealed their ability to generate sentences and human-like conversations with nuanced expressions. The advent of LLM chatbots in use has been researched for various academic tasks, such as medical examinations, and is expected to become a personal virtual assistant.

There are many potential examples of LLM applications in prevention and healthcare [24]. From the patient’s perspective, many attempts have already begun to provide virtual consultations to improve health in daily life, assist patients in deciding if they need to see a doctor, help them schedule an appointment to see a doctor, as well as explain difficult medical terms and record and consolidate health-related information. For healthcare professionals, the possibilities are great for creating medical records such as medical charts, surgical records, referral letters, discharge summaries, and handover documents, as well as for suggesting guideline-compliant treatment options and providing explanatory support to patients. It could facilitate the management, complementation, and utilization of health-related information with a high degree of personalization, scalability, and efficiency for both patients and healthcare providers. Caution should be taken before integrating these LLMs into existing healthcare systems because it is essential to address concerns about their robustness and reliability, particularly regarding the potential for probabilistically derived, non-factual discourse known as hallucination. We need to be concerned about the situation where there is no medical professional available to verify the truth or falsity of the discourse generated by LLMs. Basically, we optimistically expect that these technical shortcomings will improve day by day. Until then, it is essential to include discussions on the effective and proper use of LLMs, including technical accuracy assessments, governance to ensure a path to maximize their functionality, and remaining safeguards to ensure their safety.

## 4. How Can Artificial Intelligence Be Implemented Effectively in Diabetic Retinopathy Screening in Japan?

### 4.1. Seven Steps toward Systematic Screening of DR

**Figure 2:**
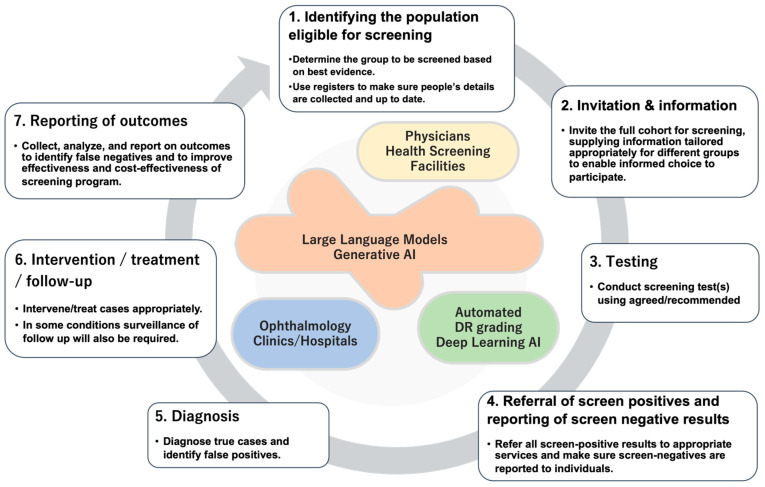
Seven steps to implement good-quality systematic screening for DR and potential cooperation with artificial intelligence (modified based on information from the Screening for Diabetic Retinopathy in Europe [5]).

The automated DR screening system has truly increased the potential to deliver good-quality screening for DR nationwide. To realize this, automated screening needs to be integrated into a well-designed screening program, and it turned out that a good-quality automated grading system does not guarantee its success. As proposed by Screening for Diabetic Retinopathy in Europe [5], the steps to establish a successful systematic screening can be broken down into seven steps (Figure 2).

### 4.2. How Will It Be Possible to Fully Utilize the Capacity of Automated Grading Systems for DR?

An automated DR screening system will surely contribute to enhancing the step of for “*Testing*” (Figure 2). By adopting automated AI-based grading as a screening test, it has the benefit of securing reproducibility, especially without concern for an inter-rater agreement. It will also speed up the turnaround from the image capture to the reporting of the results. The first pathway of DR screening in national health screening has a target population with diabetes of 1.5 to 3 million every year. To provide timely and accurate grading of the fundus images, an automated DR screening program will contribute. However, if this emerging technology is not integrated well into the screening program as a whole, it will not become effective. As shown above, there are other steps before and after the “*Testing*” step.

There is a study [25] on a demonstration of an automated AI diagnosis system for DR conducted at 11 hospitals in Thailand. They used the state-of-the-art system developed by the research group at Google and Verily. In Thailand, systematic screening for DR is conducted by taking fundus photographs of diabetic patients when they visit a physician; as the number of ophthalmologists is limited, the images are sent to the ophthalmologists, and they report the results after reviewing them. The introduction of an automated system was expected to reduce the time required to obtain test results from 2–10 weeks to 10 min. The implementation was not as expected due to multiple factors, including unstable communication infrastructure, ungradable quality images, and, above all, the human factors of the intervening screening staff and patients. It was shown that even the best-quality automated grading program for DR does not necessarily work well as a system.

### 4.3. How Will LLMs Contribute to the Screening System of DR in the Steps of Systematic Screening?

In the context of DR screening, the potential applications of LLMs can fill in the gap between the 7 steps to implement systematic screening (Figure 2). The ability to generate personalized letters can be utilized to prepare “Invitation and Information”. A personalized letter considering each person’s situation and readiness may encourage and motivate an eligible person to regularly undertake DR screening. After automated DR grading successfully processes retinal images, the grading output can be generated as a natural language so that it can achieve automated “Referral of screen positives and reporting of screen negative results”. As mentioned above, LLMs can be utilized to support “Diagnosis” and “Intervention” by supporting medical records and guideline-compliant decision-making. “Reporting of outcomes” will also be supported by LLMs through automated personalized referral letters or patient explanations.

Nosrati and Nosrati [26] illustrated the potential of AI in revolutionizing medicine not only for clinical medicine but basic science by enabling the analysis of extensive datasets, enhancing drug discovery, and optimizing biological engineering and cellular therapies. They also raised hurdles such as the scarcity of quality data for training advanced models and the complexity of capturing biological interactions. Concerns about data privacy, security, bias, and ensuring equitable access to these technologies are crucial. Collaborative efforts among researchers, policymakers, and healthcare providers are needed to establish safeguards and guidelines for AI use in this field.

## 5. Conclusions

This review tried to illustrate how screening for DR in Japan is conducted and identify what areas should be further focused on to achieve a quality systemic screening program. The use of an automated grading system with AI will boost the capacity to realize systematic screening in Japan and in other contexts by enabling fast and reproducible grading and soon replacing the primary screening task.

Despite the promising strides in integrating AI into DR screening in Japan, significant gaps persist, warranting further research. Firstly, there is a pressing need to establish unified quality standards and protocols across both screening pathways. This includes standardizing technical specifications for fundus photography and establishing rigorous training and certification programs for image evaluators. Secondly, the current lack of a dedicated information system and monitoring framework presents a major hurdle. The development of an integrated digital platform that not only facilitates the screening process but also monitors performance metrics is crucial. Furthermore, while AI technologies have shown high sensitivity and specificity in image classification, their real-world effectiveness in diverse clinical settings remains underexplored. This highlights the need for large-scale, multi-center studies to validate these technologies across various demographic and clinical scenarios. Additionally, the integration of LLMs in patient engagement and decision support is an area ripe for exploration, with a focus on addressing technical robustness and reliability, especially in preventing probabilistically derived non-factual discourse. Lastly, addressing data privacy and security concerns and ensuring equitable access to these technologies remains a key challenge. Collaborative efforts among researchers, policymakers, healthcare providers, and AI developers are essential to overcome these barriers and pave the way for a more effective, efficient, and accessible DR screening system in Japan.

*“Technology makes possibilities.*

*Design makes solutions.*

*Art makes questions.*

*Leadership makes actions”.*

—John Maeda [27]

This statement offers suggestions on how new technologies can be applied to social implementation. No matter how much potential a new technology has, it must be designed to be utilized, and leadership is required to continue to ask questions and act toward social implementation. This reemphasizes the need for ophthalmic healthcare providers to take leadership to resolve the longstanding challenge of DR screening without resting on their advantage in preventing and treating DR.

## Figures and Tables

**Table 1:** Features of organized or systematic screening that are relevant to DR screening.

Conditions Suitable for Screening	Diabetic Retinopathy
The diseases to be screened for are important and frequent.	✓ Diabetics are still increasing. ✓ Diabetic retinopathy has a risk of blindness if not treated. ✓ The social burden related to DR and vision impairment is high.
2. The disease can be easily diagnosed using non-invasive methods.	✓ Diagnosis can be made noninvasively with fundus photographs. ✓ Fundus cameras are already widely available and relatively inexpensive. ✓ Digital imaging is suitable for remote diagnosis.
3. Prevention, treatment, and rehabilitation methods after diagnosis have been established.	✓ Evidence for the treatment of diabetic retinopathy is well established. ✓ Nearsightedness can be prevented with appropriately timed treatment. ✓ Appropriate and timely treatment can also improve vision.
4. Early detection by screening leads to effective treatment after diagnosis and cost-effective	✓ Medical benefits have been shown to reduce blindness. ✓ Cost-effectiveness has been confirmed in several countries and regions, including Japan.

**Table 2:** Four stages to develop systematic screening of diabetic retinopathy (DR) (modified from information provided by the Screening for Diabetic Retinopathy in Europe [5]).

**Stage 1: Enhanced access to Effective Diabetic Retinopathy Treatment**
- Minimum number of lasers per 100,000 population - Equal access to diagnosis and treatment for all patient groups - Maximum waiting time from diagnosis to treatment (≤3 months)
**Stage 2: Establish Opportunistic Screening**
- Mydriatic fundus examination performed during routine clinic visits - Annual evaluation - National guidelines for referral to an ophthalmologist
**Stage 3: Establish systematic screening.**
- Diabetic registry to identify the target population - Systematic call-recall for registered diabetic patients - Annual fundus examination: sensitivity ≥ 80%, specificity ≥ 90%, coverage ≥ 80%.
**Stage 4: Establishment of systematic screening with complete quality assurance and coverage**
- Digital photographic screening - Trained and certified photograders - Quality assurance at all stages of the process - Data collection for monitoring and effectiveness

**Table 3:** Features of organized or systematic screening that are relevant to DR screening.

Situational Analysis for Systematic Screening	Japan (2023)
A pathway is in place, rather than the test being carried out in isolation.	Partially
The test is offered to an identified cohort of people with diabetes at an agreed-upon interval based on a register or list, rather than ad hoc offers being made or relying on individuals to request a test.	Partially
The pathway is governed by protocols and guidelines.	Partially
There are quality standards, based on evidence, that service providers follow.	No
The screening pathway is supported by an information system that can monitor performance.	No

**Table 4:** Prospective studies examining diagnostic capacity of automated screening system for DR.

Automated DR Grading System	Target DR Stages	Sensitivity	Specificity	PPV	NPV
IDx-DR x2.1 (USA, n = 819) [18]	Moderate NPDR or worse	87.2%	90.7%	-	-
	Referral DR	99.3%	68.8%	74.6%	99.1%
	Sight threatening DR	99.1%	84.0%	12%	100%
IDx-DR v2.0 (The Netherlands, n = 898) [19]	Referral DR	68.0%	86.0%	30.0%	97.0%
	Sight threatening DR	62.0%	95.0%	14.0%	99.0%
SELENA+ (Zambia, n = 1574) [20]	Referral DR	92.3%	89.0%	-	-
	Sight threatening DR	99.4%	-	-	-
	DME	97.2%	-	-	-
VoxelCloud Retina * (China, n = 15,805) [21]	Referral DR	83.3%	92.5%	61.8%	97.4%
ARDA/Verily (Thailand, n = 7517) [22]	Sight threatening DR	91.3%	96.3%	79.2%	95.5%
EyeArt v2.1 (UK, n = 30,405) [23]	Referral DR	95.7%	54.0%	-	-

DR: diabetic retinopathy; PPV: positive predictive value; NPV: negative predictive value. * non-mydriatic camera.

## Data Availability

Those who want to request data can contact the author.
